# Impact of Marine Drugs on Animal Reproductive Processes

**DOI:** 10.3390/md7040539

**Published:** 2009-11-06

**Authors:** Francesco Silvestre, Elisabetta Tosti

**Affiliations:** Animal Physiology and Evolution Laboratory, Stazione Zoologica “Anton Dohrn”, Villa Comunale, 80121-Naples, Italy

**Keywords:** marine drugs, toxins, reproduction, fertilisation, gametes

## Abstract

The discovery and description of bioactive substances from natural sources has been a research topic for the last 50 years. In this respect, marine animals have been used to extract many new compounds exerting different actions. Reproduction is a complex process whose main steps are the production and maturation of gametes, their activation, the fertilisation and the beginning of development. In the literature it has been shown that many substances extracted from marine organisms may have profound influence on the reproductive behaviour, function and reproductive strategies and survival of species. However, despite the central importance of reproduction and thus the maintenance of species, there are still few studies on how reproductive mechanisms are impacted by marine bioactive drugs. At present, studies in either marine and terrestrial animals have been particularly important in identifying what specific fine reproductive mechanisms are affected by marine-derived substances. In this review we describe the main steps of the biology of reproduction and the impact of substances from marine environment and organisms on the reproductive processes.

## 1. Introduction

### 1.1. Biology of Reproduction

Reproduction is the biological process by which new individual organisms are generated. In sexual reproduction the new organism is a combination of half of the genetic material of the two parents through the fusion of the two gametes: spermatozoon and oocyte [1]. The gametes are formed during specific processes such as oogenesis and spermatogenesis, both characterized by a unique process of cell division occurring only in gametes, called meiosis, whose goal is the production of highly specialized haploid cells for fertilisation. During gametogenesis the two gametes respectively in the ovary and the testis undergo maturation whereas gametes are activated at fertilisation. Oocyte maturation is the last phase of oogenesis during which the oocyte acquires the competence to be ovulated and fertilised. In the majority of the species, the oocyte arrests at different stages of meiotic division, in particular the block occurring in the first meiotic prophase marks the state of immature oocyte characterized by a prominent nucleus called germinal vesicle (GV). Meiosis is resumed in response to a stimulus that is different among the species and meiosis progression occurs with the germinal vesicle breakdown (GVBD). Then it progresses until a second arrest at metaphase I (MI) or II (MII) that is removed after successful fertilisation.

***Oocyte maturation*** is usually defined as the period of progression from the first to the second meiotic arrest and involves coordinated nuclear and cytoplasmic modifications [2]. If nuclear maturation is underlied by the meiotic process, cytoplasmic maturation is a more obscure process and involves both morphological and functional alterations related to: (i) changes in the expression profile of cell cycle control proteins responsible for driving the oocyte towards developmental competencies [3–7]; (ii) relocation of mitochondria and endoplasmic reticulum [8–10]; (iii) transcriptional modifications of mRNA [11]; (iv) modification of the plasma membrane permeability [12–15]; (v) differentiation of the calcium signalling machinery [16].

The control of oocyte maturation involves a complex interplay between oocyte and the extra cellular membranes and the environment, with the participation of numerous metabolic pathways. The resumption of meiotic maturation relies on two different mechanisms that are stimulation by chemical/hormonal substances and the removal of an inhibitory signal. The former involves the production of a ligand that acts on the oocyte at the GV stage inducing the GVBD.

Meiosis arrest and resumption are modulated by numerous messengers. Many studies have provided evidence of the involvement of cyclic nucleotides in the maintenance of meiotic arrest [17]. In particular, high levels of cyclic adenosine mono-phosphate (cAMP), some analogues, cAMP-dependent protein kinase (PKA) and related substances such as GPR3, act by preventing spontaneous maturation and/or blocking GVBD *in vitro* or, on the contrary, may release oocyte from meiotic arrest [18,19].

Another important factor responsible for meiotic resumption is the M-phase promoting factor (MPF) showed for the first time in amphibian oocytes in the ‘70s, by Masui [20]. Although most of the work on MPF has been carried out with frog and starfish oocytes, accumulated evidence demonstrates that this mechanism exists in other animal models, such as mammals and invertebrates [12,16,21–23].

There is a general consensus that calcium ions play a fundamental role in the resumption of meiotic maturation [4,16,24,25] in different species. The external calcium is involved in meiotic resumption in ascidian oocytes [15]. In mouse, calcium oscillations precede GVBD [26] that is delayed by removing of external calcium [27].

As showed in mammalian oocytes, meiotic maturation is a complex process that involves extensive rearrangement of microtubules and actin filaments [28]. Depending on the species, actin filaments control chromatin movement during oocyte development and regulation of organelle positioning; they also participate in oocyte cortex formation and in polarity establishment. The actin filaments, furthermore, play several roles in cortical granule movement, anchoring and exocytosis, and, together with myosin, are also involved in polar body emission [29].

***Sperm maturation*** is defined as the development of the ability of spermatozoa to fertilise eggs. In this process, the sperm undergoes morphological, biochemical, and physiological modifications initially in the testis (testicular maturation) and later in the epididymis (epididymal maturation). In the former, maturation occurs at molecular levels especially during the last phase of spermatogenesis known as spermiogenesis; here, the large round haploid spermatide undergoes a dramatic morphological and molecular changes including: replacement of histones with protamines, high condensation of chromatin, formation of the acrosome, centrioles migration and tail assemblage. In the mean time, sperm acquires a functional competence, *e.g.*, acquisition of flagellar beating providing forward propulsion and compactness of nuclear and flagellar structures.

After that, in mammals, sperm function required for fertilisation seems to be developed in the epididymis, whereas in marine animals it takes place at the moment of its spawning in the environment [1,30]. In marine organisms, spawning, i.e. the release of sperm and often eggs into the environment, is a common mechanism of reproduction [31]. The probability of successful fertilization in this mating strategy depends on many factors, including the number and the distribution of spawners [32], the timing of gamete release [33], the ways in which released gametes are dispersed [34] and finally properties of gametes themselves [35].

***Fertilisation*** is a highly specialized process of cell to cell interaction that involves many steps such as recognition of complementary receptors on the surface of the two gametes, their binding, and the fusion of the two plasma membranes [1,36]. During this complex process each gamete activates its partner. First, the spermatozoon responds to signal originating from the oocyte and its investments by rapidly changing its behaviour, form and function. Multi-step events of sperm activation involve motility, chemotaxis, first binding, acrosome reaction, second binding and fusion [1,30,37]. All these steps are mediated by known molecules. In particular, the acrosome reaction is an essential requirement to render the sperm plasma membrane highly fusible. The acrosome is an organelle located on the tip of the sperm head and due to the contact between ligand and receptors on the two gametes membranes undergoes the exocytosis [38]. This process that is calcium-mediated allows the spermatozoon to cross the zona pellucida that surrounds the oocyte and to take contact with the oocyte plasma membrane, after that fusion of the gametes occurs [29]. Following fusion, the spermatozoon triggers the quiescent oocyte into metabolic activation inducing electrical, morphological and metabolic modifications.

Electrical changes of the oocyte plasma membrane are a crucial event of the oocyte activation [40]. First indications on the role of ion currents at fertilisation were provided in marine animals in the early ‘80. In the echinoderm oocytes the occurrence of a depolarizing fertilisation potential was attributed to the activation of a transient voltage-dependent inward current [41,42]. Subsequently, it was possible to determine that the ion fluxes responsible for fertilisation potential crossed the plasma membrane as a ion current named fertilisation current [43]. Biophysical studies characterized the channels responsible for the fertilisation current as large non-specific and highly conductive ion channels [43,44] and subsequently to mainly mediated by sodium currents [14]. In marine worm eggs, the fertilisation potential can be thought of as a sum of a Na^+^-dependent “sperm receptor potential” and a superimposed Ca^2+^-dependent action potential [45].

Resting potential changes and fertilisation currents has been recorded in many vertebrate oocytes such as amphibians [46], hamster [47], rabbit [48], mouse [49], human [50] and bovine [51]. In addition, it has been shown that electrical modifications is a mechanism for preventing polyspermy in sea urchin [52], marine worm [53], ascidian [54] and Xenopus [46] oocytes.

Morphological modifications are mainly due to the triggering of the cortical reaction [9] or to the contraction of the oocyte body due to a calcium wave that crosses it from pole to pole [55].

Finally, numerous metabolic modifications occur such as modulation of phosphoinositide pathway [5–59] with inositol trisphosphate (IP_3_) formation. The latter gives rise to a fundamental event in oocyte activation [25] that is the release of intracellular calcium. This calcium signal is responsible for the exocytosis of cortical granules, resumption of meiosis and activation of development [60–65].

A calcium increase may occur in oocytes as single increase, as in amphibians [66] and sea urchins [67] or in the form of repetitive oscillations as in ascidians [68] and mammals [69–73].

In many species, fusion between sperm and the oocyte occurs at the tip of microvilli, and the actin filaments in the microvilli may participate in sperm-egg binding and fusion. As described above, the ion currents play a crucial role in the gamete activation and embryo development, so many fertilisation blockers are drugs affecting physiological modifications of cytoskeleton and/or ion currents.

In conclusion, a correct maturation and reciprocal activation of gametes are a pre-requisite for successful fertilisation and although their temporal and spatial sequences are not yet fully clarified, they involve numerous molecules in form of ligand-substrate complexes, ion current changes, and intracellular messenger pathway mobilization [24,74,75].

The basic mechanism of fertilisation has been widely debated, at present there are two main hypotheses as to how the spermatozoon triggers the oocyte into activation [24]. The first points on the binding of the spermatozoon to an oocyte membrane receptor whose signal is in turn transduced by a G-protein mechanism. The contrasting idea suggests that the spermatozoon contains a soluble factor that is released into the oocyte cytoplasm following the gamete fusion [76,77]. Both the mechanisms have been supported by experimental evidence, however in the last decade accumulating evidence indicate that the sperm factor hypothesis is more feasible. Other potential candidates were proposed by Parrington [78] to be a 33 KDa molecular mass protein and/or a truncated form of the c-kit tyrosine kinase receptor [79]. At present it has been well documented that the phospholipase Czeta is soluble factor in mammals. However, many investigators are still working to clarify the molecular nature of sperm factor in invetebrates [80,81].

***Embryo development.*** Successful fertilisation drives the oocyte into meiosis completion and exit and the formation of the zygote. This represents the first diploid cell of a new organism that divides by mitosis into a number of smaller cells named blastomeres. This process is the cleavage and is different depending on the species. Early cleavages are often synchronous, but when synchronism is lost, the blastomeres become arranged in layers or groups that mark their specific differences resulting from an unequal distribution of cytoplasmic components and/or from induction from neighboring cells. The blastomere nuclei are in fact subjected to a cytoplasmic environment that in turn affects gene activity. This triggers the programme of development that gives rise to the cell lines, e.g. the future embryo tissue (nerve, muscle, epidermis etc.). Transition from maternal gene products to the new individuals gene products takes place at different stages of division depending on the species. The first steps of embryogenesis share common characteristics among species (blastulation, gastrulation, neurulation), however the late events leading to formation of the new individual is totally different, e.g. the formation of swimming larvae and metamorphosis occurring in sea water in marine animals *vs* embryo implantation in mammals occurring *in utero*.

Although a large volume of literature deals with developmental biology, this is a very complex step-by step process that is difficult to summarize [82,83].

### 1.2. Marine Drugs

Before describing the action of marine drugs on reproductive mechanisms it is worth to mentioning that most, if not all, marine invertebrates harbour microorganisms that include bacteria, cyanobacteria and fungi within their tissues extra-and intracellular space [84,85]. The relationship between marine invertebrates and marine microorganisms that may serve as food or that live either permanently or temporarily inside of marine macroorganisms are highly complex and far from being well understood [85–87]. Microorganisms not only serve as food for filter feeders or (in the case of cyanobacteria and chemoautotrophic bacteria) enrich the diet of their hosts by carbon and nitrogen fixation, but may perhaps also be involved in the biosynthesis of natural products [88]. So, it is clear that there is often a difficulty in clarifying the real producer of a marine compound.

In this review, we have classified marine natural products in two groups: i) compounds with known and tested impact on reproduction processes; ii) compounds with plausible impact on reproductive processes, chosen on the basis of their molecular mechanisms or targets. For convenience, we describe the origin of the drugs on the basis of the zoological scale order.

#### 1.2.1. Marine Natural Products Affecting Reproduction

A limited number of studies have suggested that some of these compounds may have ecological roles as allelochemicals, specifically including compounds that may inhibit competing species. These allelochemicals may also play a role in defense against potential predators and grazers, particularly aquatic invertebrates and their larvae [89].

In the endless fight between predator and prey, the latter does not play the role of passive victim. Beyond direct defense, characterized by a series of mechanisms to avoid being killed, prey have also developed indirect long-term defenses, a sort of preventive war against predators: in fact, by producing antiproliferative compounds many organisms are able to regulate the population dynamics of their marine predators, in particular, interfering with some crucial processes of their reproductive cycle, such as: maturation, fertilisation, and early embryo development.

Cyanobacteria are Gram-negative bacteria capable of producing a wide range of potent toxins as secondary metabolites, i. e. the cyanotoxins, whose action is still rather unknown [90,91]. On the contrary, other bacteria produce widely known marine neurotoxins; **tetrodotoxin** (TTX) is one of these; it is a voltage-gated sodium current blocker and a major toxic component contained in pufferfish of the Family Tetraodontidae. Animals containing TTX are not limited to certain species of puffer. A wide variety of marine and terrestrial animals are now known to have TTX, including, but not limited to, pufferfish, salamanders, frogs, horseshoe crabs, xanthid crabs, blue-ringed octopus, and starfish [92]. In the pufferfish, TTX is concentrated in the ovary and liver, but other organs including skin, intestine, and muscle contain TTX in some species of puffer. The reason for such a wide distribution is that TTX is not produced by the puffer fish, but is produced by certain species of bacteria including *Vibrio* sp. and comes to be in the animals through the food chain [93–96]. As demonstrated in the ascidian *Ciona intestinalis*, inhibition of sodium currents at the time of fertilisation current generation gave rise to a high percentage of anomalous embryos, in which the spatial orientation at the 8/16-cell stage is lost [14].

The difficulty in identifying the real producer is clear also for another drug, **maitotoxin** (MTX), which is an extremely potent toxin obtained from the marine dinoflagellate *Gambierdiscus toxicus* and involved in ciguatera poisoning. MTX was previously detected in the viscera of maito (a small herbivorous fish, *Ctenochaetus striatus*, called “maito” in Tahiti), but further studies [97,98] found a good correlation between dinoflagellates in the gut contents and the toxicity of the viscera. The dinoflagellate turned out to represent both a new genus and new species, and it was named *Gambierdiscus toxicus* [99]. This toxin is a powerful activator of changes in the intracellular calcium concentration and it induces a potassium release from the oocytes simultaneously with a sodium entry into unfertilised eggs, although experimental evidence suggests that MTX has no ionophoretic activity *per se* [100–103]. MTX inhibits sea urchin egg fertilisation in a dose-dependent manner but, maybe, ion transport perturbations are probably not the direct cause of fertilisation inhibition which could be related to a modification of the plasma membrane of the female gametes by this hydrophilic toxin [101]. A different effect has been found in mouse, where the results suggest that putative channels activated by MTX may be involved in the calcium influx required for mouse sperm acrosome reaction [103].

Many marine drugs represent a useful tool for studying cellular processes: one of the most famous is **okadaic acid** (OA): the latter acts as a potent inhibitor of protein phosphatases [104] and has turned out to be a valuable tool for the study of phosphorylation based processes of cellular signaling [105]. The protein serine/threonine phosphatases are a unique family of enzymes that catalyze the specific dephosphorylation of phosphoserine or phosphothreonine residues in many cell types. The potent activity of OA is remarkably conserved across phyla: this toxin inhibits phosphatase activity in mammals, yeast, and higher plants [106]. OA was initially isolated and characterized from the sponges *Halichondria okadai* and *H. melanodocia* [107]. However, it was later shown to be produced by dinoflagellates of the genera *Prorocentrum* and *Dinophysis* [104,108–110] and is now considered to be of dietary origin rather than a “true” sponge metabolite in terms of its biosynthetic origin. The function of OA in sponges is not well understood. Studies by Wiens *et al.* [111] have provided evidence for at least two putative roles of OA within the sponge *Suberites domuncula*. At low concentrations, OA triggers a MAP kinase p38-regulated defense system against bacteria. At elevated concentrations, OA acts as an apoptogen and promotes expression of the proapoptotic caspase gene with a simultaneous down-regulation of the expression of the anti-apoptotic Bcl-2 homolog gene. In subsequent studies of *S. domuncula*, Schroder *et al.* [112] suggested that OA may serve as a defense molecule by inducing apoptosis in symbiotic or parasitic annelids. [113]. As protein phosphatase specific inhibitor, OA has previously been used to stimulate chromatin condensation and premature germinal vesicle breakdown in invertebrate and vertebrate oocytes [114–123].

Like TTX, **brevetoxins** are also voltage-gated sodium channel inhibitors, but these marine toxins are produced by the dinoflagellate *Karenia brevis*. Kimm-Brinson and Ramsdell [124] suggested that the larvae of medaka fish (*Oryzias latipes*), but not the eggs, are susceptible to *Karenia brevis*. In contrast, an earlier study with the sea urchin (*Lytechinus variegatus*) reported that lysates of *K. brevis* administered to eggs did induce developmental abnormalities [125]. This study found that sperm motility, egg fertilisation, and development through the blastula stage were unaffected; however, mortality and developmental abnormalities occurred in about 50% in embryos at gastrula stage and 80% in embryos at pluteus stage. The reason for the difference between the study with the red drum eggs and the sea urchin eggs may result from the use of *K. brevis* cells and lysates. The persistence of red tides from the late autumn until early spring has suggested that the spawning of some marine species may be subject to the adverse effect of red tide toxins. Steidinger *et al.* [126] also emphasized the need for attention to the effects of red tide outbreaks on migratory species, as many species seek estuaries for breeding and nursery grounds. Based on studies with other classes of fat-soluble contaminants, somatic stores of toxin in fish are transferred during oogenesis and lead to larval toxicity [127].

*K. brevis* red tides have been associated with mortality events of many aquatic animals including finfish, sea turtles, and sea birds during their adult stages [126,128–135]. These animals have been known to bioaccumulate substantial body burdens of contaminants at times, and in certain cases transfer toxicity to offspring during oogenesis. Given the similarity of developmental processes found between higher and lower vertebrates, teratogenic effects of brevetoxins have the potential to occur among different phylogenetic classes [124].

**Figure 1.**
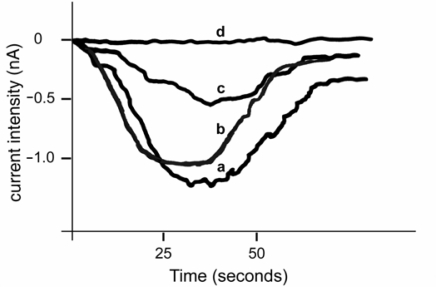
Effect of diatom-derived aldheyde 2-trans-4-trans-decadienal (DD) on fertilisation currents recorded in the whole-cell voltage clamp configuration in *Ciona intestinalis* oocytes. a: normal fertilisation current; b: oocytes incubated in acetaldehyde and then fertilised showed a normal fertilisation current similar to the control; c: oocytes incubated in the diatom aldehyde DD (1.5 μg/mL) and then fertilised showed 50% reduction in fertilisation current amplitude; d: oocytes incubated in more concentrated DD (2 μg/mL) and then fertilised showed complete inhibition of the fertilisation current. Modified from Tosti *et al.* [142].

**Figure 2.**
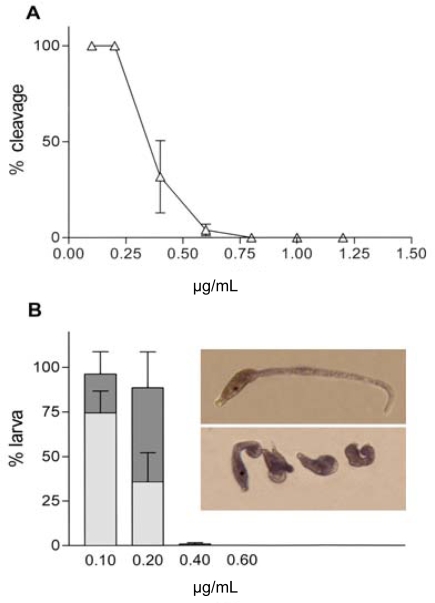
**A**: Percentage reduction of first cleavage of *Ciona intestinalis* oocytes incubated 10 minutes at different concentrations of DD and then fertilised. **B**: Percentage of embryos that reached the larval stage when exposed to different concentrations of DD. Dark shading shows the percentage of abnormal larvae. Light shading shows the percentage of normal larvae. Insert: top panel shows a normally developed *C. intestinalis* larva 24 hours after fertilisation. Bottom panel: various degrees of malformations such as stunted and elongated tail, lack of sensory organ pigmentation, blockage at the 118-cell stage (gastrula). From Tosti *et al.* [142].

The last 2 decades have seen much controversy concerning the negative impacts that consumption of diatoms may have upon copepods. Despite some contrasting data [136], diatom-derived aldehydes have been shown to interfere with reproductive mechanism in copepods [137–139] and polychaetes and echinoderms [140,141]. In the ascidian *Ciona intestinalis*, **2-*trans*-4-*trans*-decadienal** (DD) and **2-*trans*-4-*cis*-7-*cis*-decatrienal** (DT) inhibit the fertilisation current (Figure 1) which is generated in oocyte upon interaction with the spermatozoon. In particular, DD may have a dual effect on reproductive processes, influencing primary fertilisation events such as gating of fertilisation channels and secondary processes such as actin reorganization which is responsible for the segregation of cell lineages. In the same study, DD altered actin filaments and mitocondrial migration after contraction, leading to a disturbance in cleavage formation (Figure 2A). However, DD also induced larval teratogeny at low concentrations (Figure 2B), possibly due to actin perturbation [142].

**Domoic acid** (DA) is produced by red alga *Chondria armata* and planktonic diatoms of the genus *Pseudo-nitzschia*, [143]. Over the last decade, reproductive failure in California sea lions has been increasingly associated with harmful algal blooms, most notably DA produced by *Pseudo-nitzschia* spp. [144–146]. The food web plays the primary role in the transmission of DA from *Pseudo-nitzschia* blooms to the California sea lion [144,147]. Common vectors are pelagic planktivorous fish, which accumulate DA-containing diatoms in their gut exceeding toxin concentrations of one part per thousand, exceptionally high levels for a natural toxin [147,148]. Additionally, *Pseudo-nitzschia*, which form long chains of cells, will sink to the ocean floor where the DA effectively infiltrates the benthic food web and provides an additional source of vectoring [149]. At the level of the receptor in the brain, DA binds to kainate subtypes of ionotropic glutamate receptors to induce excitotoxicity by release of glutamate and activation of *N-*methyl-d-aspartate ionotropic glutamate receptors [150]. DA crosses the placenta, readily enters the neonatal brain and is retained in the amniotic fluid [151]. The early fetal brain is electrically silent, but expresses levels of ionotropic glutamate receptors that guide the migration of neurons to the appropriate brain regions and facilitate in the formation correct synapses. DA, which is normally cleared rapidly by renal filtration in adult animals, is more toxic to animals *in utero* because of a longer residence time in the fetal-maternal unit and greater access to the fetal brain [143].

The effect of DA in invertebrates is also known. As a result of the DA exposure, larval growth of king scallop, *Pecten maximus*, measured in terms of shell length and the appearance of the eye-spot, and larval survival were significantly compromised. The negative effect of DA exposure suggests that this toxin could possibly influence natural recruitment in *P. maximus*, and it may be necessary to protect hatchery-cultured scallop larvae from DA during toxic *Pseudo-nitzschia* blooms [152].

Embryo development is blocked by several drugs isolated from algae: **caulerpenyne** does not affect the microfilament-dependent processes of fertilisation and cytokinesis and allows the beginning of mitosis, but prevents normal DNA replication and results in metaphase-like arrest of sea urchin embryos [153]; **stypoldione** uncouples cytokinesis from mitosis at the lowest effective concentrations and, although it can disrupt microtubules at relatively higher concentrations, it inhibits cell division at the lowest effective concentrations by a selective action on cytokinesis through a mechanism that does not appear to involve disassembly of microtubules [154].

Sometimes it happens that the same compound may have several contrasting applications: for example, a sulfono glycolipid (**S-ACT-1**) isolated from *Gelidiella acerosa*, a Sri Lankan marine red alga, has a potent human sperm motility stimulating activity *in vitro* and has the potential to be developed into a sperm stimulant [155], but crude extracts from the same species showed elevated post-implantation loss. Since post-coital contraceptive activity of another red alga, *Gracilaria corticata,* was due to enhanced pre-implantation loss, marine red algae could represent a useful source to be harvested for potential post-coital contraceptive drugs [156].

Williamson *et al*. [157] compared the effects of chemical cues from host algae on different life history stages of the sea urchin *Holopneustes purpurascens*. In sublittoral habitats, *H. purpurascens* occurred primarily on two algal hosts: red alga (*Delisea pulchra*) and kelp (*Ecklonia radiata*). Sea urchin larvae rapidly metamorphosed in the presence of *D. pulchra*, but metamorphosis was delayed or absent in the presence of *E. radiata. D. pulchra* produces a polar chemical inducer of metamorphosis not found in *E. radiata*. In contrast to larval metamorphosis, feeding and performance of juvenile and adult sea urchins were considerably worse on *D. pulchra* than on *E. radiata*.

To our knowledge, there are just few marine drugs that affect gamete maturation by interacting with the cytoskeleton [158–160]: among them, **jasplakinolide**, a marine compound from sponges, was found to arrest oocytes *in vitro* maturation acting as a microfilament inhibitor, this also affects fertilisation in mice [161,162]; **theonellapeptolide Ie**, from *Petrosa* species, induced malformed maturation through disturbance of cortical F-actin distribution [158]; also **strongylophorines**, isolated from *Strongylophora strongylata*, inhibited the maturation of starfish oocytes by affecting actin [160].

Other marine toxins, the **latrunculins**, produced by certain sponges, including genus *Latrunculia*, inhibit the microfilament-mediated processes during fertilisation, cleavage and early development in sea urchins and mice [163], even more potent than the cytochalasins [164].

However, not all the compounds from the sponge have the same target: for example, **(−)-10-epi-axisonitrile-3**, a spirocyclic sesquiterpene isocyanide obtained from the marine sponge *Geodia exigua*, immobilized sperm of sea urchin and starfish and in turn block fertilisation, by inhibiting the phosphocreatine shuttle participating in the high-energy phosphate metabolism [165]; **theonellapeptolide IId**, isolated from *Theonella swinhoei*, prevented fertilisation of sea urchin while has no effect on early embryonic development of fertilised eggs [166]; also **jaspisin**, isolated from the extract of a marine sponge, *Jaspis* species, has different properties: it is a selective inhibitor of exoplasmic membrane fusion in echinoderms, blocking fusion between the sperm acrosomal process and the egg plasma membrane, and also embryo hatching [167–169]. Similarly to jaspisin, but differently from 10-epi-Axisonitrile-3**,** there are other inhibitors of echinoderm fertilisation isolated from sponge, **callyspongins A and B** [170] and **halenaquinol sulfate** [171,172], that are able to inhibit sperm-egg fusion without affecting sperm motility [165]. **Calyculin-A**, originally derived from the marine sponge *Discodermia calyx*, is similar to okadaic acid in its potent inhibition of protein phosphatases and thus in its impact on reproduction [173,174].

Bacteria, algae and sponges are not the only source of marine products: Agius *et al.* [175] demonstrated that under illumination **bonellin,** a compound isolated from *Bonellia viridis* [176], causes depression of oxygen uptake by spermatozoa and developmental arrest of echinoid and Bonellia eggs. The effect on the eggs may primarily be due to the crosslinking of membrane proteins and to the formation of peroxydase that results in the cytolysis of the unfertilised eggs and in the inhibition of cleavage [177]. Bonellin is able to arrest the motility of swimming larvae of *C. intestinalis*, but, since the fibrillar structures appear to be unaltered, also this impact seems a consequence of membrane modifications [178].

Recently, spermicidal activity has been found in sea cucumber *Bohadschia vitiensis* whole-body extracts followed by isolation and characterization of bioactive molecule, **bivittoside D**, able to induce human sperm membrane permeabilization [179].

Even some early chordates are supposed to be a promising source for marine drugs affecting reproduction processes: **methoxyconidiol**, extracted from the ascidian *Aplidium aff. densum* [180], inhibits the cleavage of sea urchin *Sphaerechinus granularis* and *Paracentrotus lividus* fertilised eggs: infact, it disrupts M-phase progression and completely blocks cytokinesis without having any effect on DNA replication, most likely by affecting microtubule dynamics [181]. In Table 1, we provide a summary of the significant data described above.

#### 1.2.2. Potential Impact of Marine Drugs

As described above, reproduction is a complex process characterized by many crucial steps. It is clear that every marine compound able to affect in some way one of these events could affect reproduction either in natural setting and *in vitro*. A good example to better understand the huge potential of marine organisms as drug source is given by marine sponges. The latter have been considered to be a gold mine during the past 50 years, with respect to the diversity of their secondary metabolites [182]. A huge amount of marine products has been described [183–187] and sponges, in particular, are the source of more than 5,300 different products. Every year hundreds of new compounds are being discovered [184–186], leading to identification of novel therapeutic agents showing a broad spectrum of pharmacological activity; therefore, more marine natural products will probably become potential leads for clinical development as novel therapeutic agents for the treatment of multiple disease categories [188–190].

Marine compounds showed different effects on many molecules that are in different ways involved in reproduction, for example, they inhibit protein kinase C [113,191–193], calcium [194–196], sodium [197–201] and potassium channels [202–206]. Similarly, other targets are IP_3_ receptors, endoplasmic reticulum calcium pumps [207], microtubules [208–216]; microfilaments [217–221]; and plasma membrane [222,223].

Even though this is not proved yet, we may hypothesize their plausible impact on reproduction on the basis of their molecular targets, either *in vivo* and *in vitro*. Over the last few decades significant efforts have been made, by both pharmaceutical companies and academic institutions, to isolate and identify new marine-derived natural products. Because of the diversity in ocean life many new potentially interesting compounds are continuously being discovered.

Despite the risks associated with marine toxins, such as food poisoning or harmful algal blooms, these biogenic compounds have proven their advantage and potential in several fields, particularly as new therapeutic agents for a variety of diseases: in fact, marine ecosystem is an enormously rich source of natural products with promising therapeutic usefulness in oncology, providing anticancer agents with novel mechanisms of action [224–227].

This review reports current understanding of the impacts of compounds from marine organisms on the main reproductive processes and some of their specific mechanisms underlying gametogenesis, fertilisation and very early embryo development. Although most of the data provide evidence that such compounds adversely affect reproductive success, marine drugs may represent a wide source with potential application in improving reproductive fitness.

## Figures and Tables

**Table 1.** Summary of significant data reported in the text.

**Drug**	**Source**	**Target - Impact**	**References**
Tetrodotoxin	Bacteria including *Vibrio* sp.	- Voltage-gated sodium channels; - Inhibition of fertilisation current in *C. intestinalis;* teratogenic effect	[14,96,200]
Maitotoxin	Dinoflagellate *Gambierdiscus toxicus*	- Cationic channel - Mouse acrosome reaction; inhibition of sea urchin egg fertilisation	[101,103]
Okadaic acid	Dinoflagellates *Prorocentrum lima* and *Dinophysis* spp.	- Protein phosphatases; - Inhibition of phosphorylation based processes of cellular signaling	[104,123]
Brevetoxin	Dinoflagellate *Karenia brevis*	- Voltage-gated sodium channels; - Teratogenic effect	[124]
2-*trans*,4-*trans* Decadienal	Diatoms	- Fertilisation current; - Inhibition of embryonic development and fertilisation in broadcast spawning marine invertebrates	[140,142]
Domoic acid	Alga *Chondria armata* Diatom *Pseudonitzschia*	- Ionotropic glutamate receptors; - Neurotoxicity; reduction of larval growth and survival in *P. maximus* ; reproductive failure in California sea lions.	[143,152]
Caulerpenyne	Alga *Caulerpa taxifolia*	- Microtubules; - Inhibition of first cell division	[153]
Stypoldione	Alga Stypopodium zonale	- Sulfhydryl groups of proteins - Inhibition of cytokinesis in sea urchin embryos	[154]
Sulfonoglycolipid S-ACT-1	Alga *Gelidiella acerosa*	- Sperm - Stimulation of sperm motility	[155]
Crude extract	Alga *Gracilaria corticata,*	- Increase of pre-implantation loss in femal rats	[156]
Crude extract	Alga *Gelidiella acerosa*	- Increase of post-implantation loss in female rats	[156]
Jasplakinolide	Sponge *Jaspis johnstoni*	- Actin; - Arrest of *in vitro* maturation	[161,162]
Theonellapeptolide Ie	Sponge *Petrosia*	- Cortical F-actin distribution; - Abnormal maturation	[158]
Strongylophorine	Sponge *Strongylophora strongylata*	-Actin; -Inhibition of the maturation of starfish oocytes	[160]
Latrunculin	Sponge *Latrunculia magnifica*	- Microfilament; - Inhibition of microfilament-mediated processes during fertilisation, cleavage and early development in sea urchins and mice.	[163]
(−)-10-*epi*-Axiso-nitrile-3	Sponge *Geodia Exigua*	- Phosphocreatine shuttle; - Sea urchin and starfish sperm immobilization	[165]
Theonellapeptolide IId	Sponge *Theonella swinhoei*	- Inhibition of fertilisation of the sea urchin	[166]
Jaspisin	Sponge *Jaspis* sp.	- Exoplasmic membrane fusion; - Block fusion between sperm acrosomal process and egg plasma membrane; block of embryo hatching	[167–169]
Callyspongins A and B	Sponge *Callyspongia truncata*	- Inhibition of sperm-egg fusion	[170]
Halenaquinol sulfate	Sponge *Xestospongia sapra*	- Inhibition of sperm-egg fusion	[171,172]
Calyculin A	Sponge *Discodermia calyx*	- Protein phosphatases - Modulation of phosphorylation based processes of cellular signalling	[174]
Bonellin	Echiura *Bonellia viridis*	- Membrane proteins; - Formation of peroxydase; cytolysis of unfertilised eggs, inhibition of cleavage and of larval motility	[178]
Bivittoside D	Sea cucumber *Bohadschia vitiensis*	- Sperm; - Membrane permeabilization; spermicide activity in human	[179]
Methoxyconidiol	Ascidian *Aplidium aff. densum*	- Microtubules; - Inhibition of cleavage	[180,181]

## References

[1] Yanagimachi R Mammalian fertilization The Physiology of Reproduction Knobil E Neil J Raven Press New York, NY, USA 1994 1 189 317

[2] Eppig JJ Coordination of nuclear and cytoplasmic oocyte maturation in eutherian mammals Reprod Fertil Dev 1996 8 485 489 8870074 10.1071/rd9960485

[3] Masui Y A quest for cytoplasmic factors that control the cell cycle Prog Cell Cycle Res 1996 2 1 13 9552378 10.1007/978-1-4615-5873-6_1

[4] Whitaker M Patel R Calcium and cell cycle control Development 1990 108 525 542 2167196 10.1242/dev.108.4.525

[5] Whitaker M Control of meiotic arrest Rev Reprod 1996 1 127 135 9414449 10.1530/ror.0.0010127

[6] Kishimoto T Cell-cycle control during meiotic maturation Curr Opin Cell Biol 2003 15 654 663 14644189 10.1016/j.ceb.2003.10.010

[7] Haccard O Jessus C Oocyte maturation, Mos and cyclins-A matter of synthesis: Two functionally redundant ways to induce meiotic maturation Cell Cycle 2006 5 1152 1159 16760654 10.4161/cc.5.11.2800

[8] Ducibella TA Anderson DF Albertini F Aalberg J Rangarajan S Quantitative studies of changes in cortical granule number and distribution in the mouse oocyte during maturation Dev Biol 1988 130 184 197 3141231 10.1016/0012-1606(88)90425-3

[9] Wessel GM Brooks JM Green E Haley S Voronina E Wong J Zaydfudim V Conner S The biology of cortical granules Int Rev Cytol 2001 209 117 206 11580200 10.1016/s0074-7696(01)09012-x

[10] Prodon F Chenevert J Sardet C Establishment of animal-vegetal polarity during maturation in ascidian oocytes Dev Biol 2006 290 297 311 16405883 10.1016/j.ydbio.2005.11.025

[11] Hake LE Richter JD Translational regulation of maternal mRNA Biochem Biophys Acta 1997 1332 31 38 10.1016/s0304-419x(96)00039-x 9061009

[12] Carroll J Na^+^-Ca^2+^ exchange in mouse oocytes: Modifications in the regulation of intracellular free Ca^2+^ during oocyte maturation J Reprod Fert 2000 118 337 342 10.1530/jrf.0.1180337 10864798

[13] Cuomo A Di Cristo C Di Cosmo A Paolucci M Tosti E Calcium currents correlate with oocyte maturation during the reproductive cycle in Octopus vulgaris J Exp Zool A 2005 303 193 202 10.1002/jez.a.152 15726628

[14] Cuomo A Silvestre F De Santis R Tosti E Ca^2+^ and Na^+^ current patterns during oocyte maturation, fertilization, and early developmental stages of *Ciona intestinalis* Mol Reprod Dev 2006 73 501 511 16425233 10.1002/mrd.20404

[15] Silvestre F Cuomo A Tosti E Ion current activity and molecules modulating maturation and growth stages of ascidian (*Ciona intestinalis*) oocytes Mol Repodr Dev 2009 76 1084 1093 10.1002/mrd.21073 19565642

[16] Homa S Calcium and meiotic maturation of the mammalian oocyte Mol Reprod Dev 1995 40 122 134 7702866 10.1002/mrd.1080400116

[17] Gilchrist R Thompson JG Oocyte maturation: Emerging concepts and technologies to improve developmental potential in vitro Theriogenology 2007 67 6 15 17092551 10.1016/j.theriogenology.2006.09.027

[18] Richard FJ Regulation of meiotic maturation J Animal Sci 2007 85 E4 E6 10.2527/jas.2006-475 17040950

[19] Vaccari S Horner K Mehlmann LM Conti M Generation of mouse oocytes defective in cAMP synthesis and degradation: Endogenous cyclic AMP is essential for meiotic arrest Dev Biol 2008 316 124 134 18280465 10.1016/j.ydbio.2008.01.018 PMC2755085

[20] Masui Y From oocyte maturation to the in vitro cell cycle: The history of discoveries of Maturation-Promoting Factor (MPF) and Cytostatic Factor (CSF) Differentiation 2001 69 1 17 11776390 10.1046/j.1432-0436.2001.690101.x

[21] Russo GL Kyozuka K Antonazzo L Tosti E Dale B Maturation promoting factor in ascidian oocytes is regulated by different intracellular signals at meiosis I and II Development 1996 122 1995 2003 8681780 10.1242/dev.122.7.1995

[22] Kishimoto T Cell cycle arrest and release in starfish oocytes and eggs Semin Cell Dev Biol 1998 9 549 557 9835643 10.1006/scdb.1998.0249

[23] Yamashita M Mita K Yoshida N Kondo T Molecular mechanisms of the initiation of oocyte maturation: General and species-specific aspects Prog Cell Cycle Res 2000 4 115 129 10740820 10.1007/978-1-4615-4253-7_11

[24] Tosti E Calcium ion currents mediating oocyte maturation events Reprod Biol Endocrinol 2006 4 26 16684344 10.1186/1477-7827-4-26 PMC1475868

[25] Whitaker M Calcium at fertilization and in early development Physiol Rev 2006 8625 88 10.1152/physrev.00023.2005 PMC3299562 16371595

[26] Carroll J Swann K Whittingham D Whitaker M Spatiotemporal dynamics of intracellular [Ca^2+^]_i_ oscillations during the growth and meiotic maturation of mouse oocytes Development 1994 120 3507 3517 7821218 10.1242/dev.120.12.3507

[27] Tombes RM Simerly C Borisy GG Schatten G Meiosis, egg activation, and nuclear envelope breakdown are differentially reliant on Ca^2+^, whereas germinal vesicle breakdown is Ca^2+^ independent in the mouse oocyte J Cell Biol 1992 117 799 811 1577859 10.1083/jcb.117.4.799 PMC2289470

[28] Roth Z Hansen PJ Disruption of nuclear maturation and rearrangement of cytoskeletal elements in bovine oocytes exposed to heat shock during maturation Reproduction 2005 129 235 244 15695618 10.1530/rep.1.00394

[29] Sun QY Schatten H Regulation of dynamic events by microfilaments during oocyte maturation and fertilization Reproduction 2006 131 193 205 16452714 10.1530/rep.1.00847

[30] Tosti E Sperm activation in species with external fertilization Zygote 1994 2 359 361 8665169

[31] Stratmhann RR Why life histories evolve differently in the sea Soc Integ Comp Biol 1990 30 197 207

[32] Levitan DR Young CM Reproductive success in large populations: Empirical measures and theoretical predictions of fertilization in the sea biscuit *Clypeaster rosaceus* J Exp Mar Biol Ecol 1995 190 221 241

[33] Sewell MA Levitan DR Fertilization success during a natural spawning of the dendrochirote sea cucumber Cucumaria miniata Bull Mar Sci 1992 51 161 166

[34] Mead KS Denny MW The effects of hydrodynamic shear stress on fertilization and early development of the purple sea urchin *Strongylocentrotus purpuratus* Biol Bull 1995 188 46 56 7696387 10.2307/1542066

[35] Thomas F Physical properties of gametes in three sea urchin species J Exp Biol 1994 194 263 284 9317771 10.1242/jeb.194.1.263

[36] Dale B Fertilization in animals Arnold Edward The Camelot Pres Ltd London, UK 1983

[37] Aitken RJ Sperm function tests and fertility Int J Androl 2006 29 69 75 16466526 10.1111/j.1365-2605.2005.00630.x

[38] Breitbart H Signaling pathways in sperm capacitation and acrosome reaction Cell Mol Biol 2003 49 321 327 12887084

[39] Stein KK Primakoff P Myles D Sperm-egg fusion: Events at the plasma membrane J Cell Sci 2004 1117 6269 6274 15591242 10.1242/jcs.01598

[40] Tosti E Boni R Electrical events during gamete maturation and fertilisation in animals and human Hum Reprod Update 2004 10 53 65 15005464 10.1093/humupd/dmh006

[41] Dale B DeFelice LJ Taglietti V Membrane noise and conductance increase during single spermatozoon-egg interaction Nature 1978 275 217 219 29229 10.1038/275217a0

[42] Dale B de Santis A Maturation and fertilization of the sea urchin oocyte: An electrophysiological study Dev Biol 1981 85 474 484 6790323 10.1016/0012-1606(81)90278-5

[43] Dale B DeFelice LJ Sperm-activated channels in ascidian oocytes Dev Biol 1984 101 235 239 6319209 10.1016/0012-1606(84)90135-0

[44] DeFelice LJ Kell MJ Sperm-activated currents in ascidian oocytes Dev Biol 1987 119 123 128 3792625 10.1016/0012-1606(87)90213-2

[45] Jaffe LA Gould-Somero M Holland L Ionic mechanism of the fertilization potential of the marine worm, *Urechis caupo* (Echiura) J Gen Physiol 1979 73 469 492 571895 10.1085/jgp.73.4.469 PMC2215169

[46] Glahn D Nuccitelli R Voltage-clamp study of the activation currents and fast block to polyspermy in the egg of *Xenopus laevis* Dev Growth Diff 2003 45 187 197 10.1034/j.1600-0854.2004.00684.x 12752506

[47] Miyazaki S Igusa Y Fertilization potential in golden hamster eggs consists of recurring hyperpolarization Nature 1981 290 702 704 6894326 10.1038/290702a0

[48] McCulloh DH Rexroad CE Jr Levitan H Insemination of rabbit eggs is associated with slow depolarization and repetitive diphasic membrane potentials Dev Biol 1983 95 372 377 6687462 10.1016/0012-1606(83)90038-6

[49] Igusa Y Miyazaki S Yamashita N Periodic hyperpolarizing responses in hamster and mouse eggs fertilized with mouse sperm J Physiol 1983 340 633 647 6411906 10.1113/jphysiol.1983.sp014784 PMC1199231

[50] Gianaroli L Tosti E Magli C Iaccarino M Ferraretti AP Dale B Fertilization current in the human oocyte Mol Reprod Dev 1994 38 209 214 8080650 10.1002/mrd.1080380212

[51] Tosti E Boni R Cuomo A Fertilization and activation currents in bovine oocytes Reproduction 2002 124 835 846 12530921

[52] Jaffe LA Fast block to polyspermy in sea urchin eggs is electrically mediated Nature 1976 261 68 71 944858 10.1038/261068a0

[53] Gould-Somero M Jaffe LA Holland LZ Electrically mediated fast polyspermy block in eggs of the marine worm Urechis caupo J Cell Biol 1979 82 426 440 10.1083/jcb.82.2.426 PMC2110452 39082

[54] Goudeau H Depresle Y Rosa A Goudeau M Evidence by a voltage clamp study of an electrically mediated block to polyspermy in the egg of the ascidian *Phallusia mammillata* Dev Biol 1994 166 489 501 7813772 10.1006/dbio.1994.1332

[55] Satoh N Developmental Biology of Ascidians Cambridge University Press New York, NY, USA 1994

[56] Turner PR Jaffe LA Fein A Regulation of cortical vesicle exocytosis in sea urchin eggs by inositol 1,4,5-trisphosphate and GTP-binding protein J Cell Biol 1986 102 70 76 3001104 10.1083/jcb.102.1.70 PMC2114041

[57] Stith BJ Espinoza R Roberts D Smart T Sperm increase inositol 1,4,5-trisphosphate mass in *Xenopus laevis* eggs preinjected with calcium buffers or heparin Dev Biol 1994 165 206 215 8088439 10.1006/dbio.1994.1247

[58] Dupont G McGuinness OM Johnson MH Berridge MJ Borgese F Phospholipase C in mouse oocytes: Characterization of beta and gamma isoforms and their possible involvement in sperm-induced Ca^2+^ spiking Biochem J 1996 316 583 591 8687404 10.1042/bj3160583 PMC1217388

[59] Lee SJ Madden PJ Shen SS U73122 blocked the cGMP-induced calcium release in sea urchin eggs Exp Cell Res 1998 242 328 334 9665830 10.1006/excr.1998.4070

[60] Kline D Kline JT Repetitive calcium transients and the role of calcium in exocytosis and cell cycle activation in the mouse egg Dev Biol 1992 149 80 89 1728596 10.1016/0012-1606(92)90265-i

[61] Miyazaki S Shirakawa H Nakada K Honda Y Essential role of the inositol 1,4,5-trisphosphate receptor/Ca^2+^ release channel in Ca^2+^ waves and Ca^2+^ oscillations at fertilization of mammalian eggs Dev Biol 1993 158 62 78 8392472 10.1006/dbio.1993.1168

[62] Swann K Ozil JP Dynamics of the calcium signal that triggers mammalian egg activation Int Rev Cytol 1994 152 183 222 8206704 10.1016/s0074-7696(08)62557-7

[63] Xu Z Kopf GS Schultz RM Involvement of inositol 1,4,5-trisphosphate-mediated Ca^2+^ release in early and late events of mouse egg activation Development 1994 120 1851 1859 7924992 10.1242/dev.120.7.1851

[64] Abbott AL Ducibella T Calcium and the control of mammalian cortical granule exocytosis Front Biosci 2001 6 D792 D806 11438440 10.2741/abbott

[65] Malcuit C Kurokawa M Fissore RA Calcium oscillations and mammalian egg activation J Cell Physiol 2006 206 565 573 16155907 10.1002/jcp.20471

[66] Busa WB Nuccitelli R An elevated free cytosolic Ca^2+^ wave follows fertilization in eggs of the frog, *Xenopus laevis* J Cell Biol 1985 100 1325 329 3980584 10.1083/jcb.100.4.1325 PMC2113751

[67] Jaffe LA Giusti AF Carroll DJ Foltz KR Ca^2+^ signalling during fertilization of echinoderm eggsseminars in Cell & Dev. Biol 2001 12 45 51 10.1006/scdb.2000.0216 11162746

[68] Dumollard R McDougall A Rouvière C Sardet C Fertilisation calcium signals in the ascidian egg Biol Cell 2004 96 29 36 15093125 10.1016/j.biolcel.2003.11.002

[69] He CL Damiani P Parys JB Fissore RA Calcium, calcium release receptors, and meiotic resumption in bovine oocytes Biol Reprod 1997 57 1245 1255 9369194 10.1095/biolreprod57.5.1245

[70] Mehlmann LM Kline D Regulation of intracellular calcium in the mouse egg: Calcium release in response to sperm or inositol trisphosphate is enhanced after meiotic maturation Biol Reprod 1994 51 1088 1098 7888488 10.1095/biolreprod51.6.1088

[71] Ducibella T Huneau D Angelichio E Xu Z Schultz RM Kopf GS Fissore RA Madoux S Ozil JP Egg-to-embryo transition is driven by differential responses to Ca^2+^ oscillation number Dev Biol 2002 250 280 291 12376103

[72] Miyazaki S Ito M Calcium signals for egg activation in mammals J Pharmacol Sci 2006 100 545 552 16799264 10.1254/jphs.cpj06003x

[73] Swann K Yu Y The dynamics of calcium oscillations that activate mammalian eggs Int J Dev Biol 2008 52 585 594 18649272 10.1387/ijdb.072530ks

[74] Dale B Oocyte activation in invertebrates and humans Zygote 1994 2 373 377 8665173 10.1017/s0967199400002252

[75] Runft LL Jaffe LA Mehlmann LM Egg activation at fertilization: Where it all begins Dev Biol 2002 245 237 54 11977978 10.1006/dbio.2002.0600

[76] Wilding M Dale B Sperm factor: What is and what I does it do? Mol Human Reprod 1997 3 269 273 10.1093/molehr/3.3.269 9237253

[77] Swann K Larman MG Saunders CM Lai FA The cytosolic sperm factor that triggers Ca^2+^ oscillations and egg activation in mammals is a novel phospholipase C: PLCzeta Reproduction 2004 127 431 439 15047934 10.1530/rep.1.00169

[78] Parrington J Swann K Shevchenko VI Sesay AK Lai FA Calcium oscillations in mammalian eggs triggered by a soluble sperm protein Nature 1996 379 364 368 8552195 10.1038/379364a0

[79] Sette C Bevilacqua A Bianchini A Mangia F Geremia R Rossi P Parthenogenetic activation of mouse eggs by microinjection of a truncated c-kit tyrosine kinase present in spermatozoa Development 1997 124 2267 2274 9187152 10.1242/dev.124.11.2267

[80] Saunders CM Larman MG Parrington J Cox LJ Royse J Blayney LM Swann K Lai FA PLC zeta: A sperm-specific trigger of Ca(2+) oscillations in eggs and embryo development Development 2002 129 3533 3544 12117804 10.1242/dev.129.15.3533

[81] Tosti E Menezo Y Sperm induced oocyte activation Human Spermatozoa: Maturation, Capacitation and Abnormalities Nova Science Publishers Hauppage, NY, USA, in press.

[82] Alberts B Bray D Lewis J Raff M Roberts K Watson JD Cellular mechanisms of development Molecular Biology of the Cell Garland Publishing New York, NY, USA 1983 813 890

[83] Menezo Y Renard JP The life of egg before implantation Reproduction in Mammals and Man Thibault C Levassouf MC AHF Unter Elipses Paris, France 1993 350 366

[84] Vacelet J Donadey C Electron microscope study of the association between some sponges and bacteria J Exp Mar Ecol 1977 30 301 314

[85] Wilkinson CR Symbiotic interactions between marine sponges and algae Algae and Symbioses Reisser W Biopress Bristol, UK 1992 112 151

[86] Hentschel U Steinert M Hacker J Common molecular mechanisms of symbiosis and pathogenesis Trends Microbiol 2000 8 226 231 10785639 10.1016/s0966-842x(00)01758-3

[87] Steinert M Hentschel U Hacker J Symbiosis and pathogenesis: Evolution of the microbe-host interaction Naturwissenschaft 2000 7 1 11 10.1007/s001140050001 10663126

[88] Proksch P Edrada RA Ebel R Drugs from the seas - Current status and microbiological implications Appl Microbiol Biotechnol 2002 59 125 134 12111137 10.1007/s00253-002-1006-8

[89] Berry JP Gantar M Perez MH Berry G Noriega FG Cyanobacterial toxins as allelochemicals with potential applications as algaecides, herbicides and insecticides Mar Drugs 2008 6 117 146 18728763 10.3390/md20080007 PMC2525484

[90] van Apeldoorn ME van Egmond HP Speijers GJ Bakker GJ Toxins of cyanobacteria Mol Nutr Food Res 2007 51 7 60 17195276 10.1002/mnfr.200600185

[91] Duy TN Lam PKS Shaw G Connell DW Toxicology and risk assessment of freshwater cyanobacterial (bluegreen algal) toxins in water Rev Environ Contam Toxicol 2000 163 113 186 10771585 10.1007/978-1-4757-6429-1_3

[92] Miyazawa K Noguchi T Distribution and origin of tetrodotoxin J ToxicolTox Rev 2001 20 11 33

[93] Noguchi T Jeon JK Arakawa O Sugita H Deguchi Y Shida Y Hashimoto K Occurrence of Tetrodotoxin and Anhydrotetrodotoxin in *Vibrio* sp. Isolated from the Intestines of a Xanthid Crab, *Atergatis floridus* J Biochem 1986 99 311 314 3754255 10.1093/oxfordjournals.jbchem.a135476

[94] Narita H Matsubara S Miwa N Akahane S Murakami M Goto T Nara M Noguchi T Saito T Shida Y Hashimoto K *Vibrio alginolyticus*, a TTX-producing bacterium isolated from the starfish *Astropecten polyacanthus* Bull Japan Soc Sci Fish 1987 47 935 941

[95] Hashimoto K Noguchi T Watabe S Microbial Toxins in Foods and Feeds Pohland AE Dowell VR Jr Richard JL Plenum Press New York, NY, USA 1990 159 172

[96] Naharashi T Tetrodotoxin: A brief history Proc Jpn Acad Ser B Phys Biol Sci 2008 84 147 154 10.2183/pjab.84.147 PMC2858367 18941294

[97] Yasumoto T Murata M Marine toxins Chem Rev 1993 93 1897 1909

[98] Yasumoto T Satake M Chemistry, Etiology and Determination Methods of Ciguatera Toxins J Toxicol Tox Rev 1996 15 91 107

[99] Yasumoto T The Chemistry and Biological Function of Natural Marine Toxins Chem Rec 2001 1 228 242 11895121 10.1002/tcr.1010

[100] Takahashi M Ohizumi Y Yasumoto T Maitotoxin, a Ca^2+^ channel activator candidate J Biol Chem 1982 257 7287 7289 6282837

[101] Pesando D Girard JP Durand-Clément M Payan P Puiseux-Dao S Effect of maitotoxin on sea urchin egg fertilization and on Ca^2+^ permeabilities of eggs and intracellular stores Biol Cell 1991 72 269 273 1794068 10.1016/0248-4900(91)90297-z

[102] Escobar LI Salvador C Martinez M Vaca L Maitotoxin, a cationic channel activator Neurobiology 1998 6 59 74 9713832

[103] Treviño CL De la Vega-Beltrán JL Nishigaki T Felix R Darszon A Maitotoxin potently promotes Ca^2+^ influx in mouse spermatogenic sells and sperm, and snduces the acrosome reaction J Cell Physiol 2006 206 449 456 16155908 10.1002/jcp.20487

[104] Yasumoto T Seino N Murakami Y Murata M Toxins produced by benthic dinoflagellates Biol Bull 1987 172 128 131

[105] Dounay AB Forsyth CJ Okadaic acid: The archetypal serine/threonine protein phosphatase inhibitor Curr Med Chem 2002 9 1939 1980 12369865 10.2174/0929867023368791

[106] Cohen P Holmes CFB Tsukitani Y Okadaic acid: A new probe for studying cellular regulation Trends Biochem Sci 1990 15 98 102 2158158 10.1016/0968-0004(90)90192-e

[107] Tachibana K Scheuer P Tsukitani Y Kikuchi H van Engen D Clardy J Gopichand Y Schmitz FJ Okadaic acid, a cytotoxic polyether from two marine sponges of the genus *Halichondria* J Am Chem Soc 1981 103 2469 2471

[108] Murakami Y Oshima Y Yasumoto T Identification of okadaic acid as a toxic component of a marine dinoflagellate Prorocentrum lima Bull Jpn Soc Sci Fish 1982 48 69 72

[109] Zhou J Fritz L Okadaic acid antibody localizes to chloroplasts in the DSP-toxin-producing dinoflagellates *Prorocentrum lima* and *Prorocentrum maculosum* Phycologia 1994 33 455 461

[110] McLachlan JL Marr JC Conlon-Kelly A Adamson A Effects of nitrogen concentration and cold temperature on DSP-toxin concentrations in the dinoflagellate *Prorocentrum lima* (Prorocentrales, Dinophyceae) J Nat Toxins 1994 2 263 270 10.1002/nt.2620020504 7866661

[111] Wiens M Luckas B Brümmer F Shokry M Ammar A Steffen R Batel R Diehl-Seifert B Schröder HC Müller WEG Okadaic acid: A potential defense molecule for the sponge *Suberites domuncula* Mar Biol 2003 142 213 223

[112] Schröder HC Breter HJ Fattorusso E Ushijima H Wiens M Steffen R Batel R Müller WEG Okadaic acid, an apoptogenic toxin for symbiotic/parasitic annelids in the demosponge Suberites domuncula Appl Environ Microbiol 2006 72 4907 4916 10.1128/AEM.00228-06 PMC1489365 16820487

[113] Paul VJ Arthur KE Ritson-Williams R Ross C Sharp K Chemical defenses: From compounds to communities Biol Bull 2007 213 226 251 18083964 10.2307/25066642

[114] Pondaven P Cohen P Identification of protein phosphatases-l and 2A and inhibitor-2 in oocytes of the starfish *Asterias rubens* and *Marthasterias glacialis* Eur J Biochem 1987 167 135 140 3040398 10.1111/j.1432-1033.1987.tb13314.x

[115] Goris J Hermann J Hendrix P Ozon R Merlevede W Okadaic acid, a specific protein phosphatase inhibitor, induces maturation and MPF formation in *Xenopus laevis* oocytes FEBS Lett 1989 245 91 94 2538367 10.1016/0014-5793(89)80198-x

[116] Rime H Ozon R Protein phosphatases are involved in the in vivo activation of histone HI kinase in mouse oocyte Dev Biol 1990 141 115 122 2167856 10.1016/0012-1606(90)90106-s

[117] Alexandre H van Cauwenberge A Tsukitani Y Mulnard J Pleiotropic effect of okadaic acid on maturing mouse oocytes Development 1991 112 971 980 1718679 10.1242/dev.112.4.971

[118] Gavin AC Tsukitani Y Schorderet-Slatkine S Induction of M-phase entry of prophase-blocked mouse oocytes through microinjection of okadaic acid, a specific phosphatase inhibitor Exp Cell Res 1991 192 75 81 1701730 10.1016/0014-4827(91)90159-r

[119] Schwartz DA Schultz RM Stimulatory effect of okadaic acid, an inhibitor of protein phosphatases, on nuclear envelope breakdown and protein phosphorylation in mouse oocytes and one-cell embryos Dev Biol 1991 145 119 127 1850367 10.1016/0012-1606(91)90218-r

[120] Gavin AC Vassalli JD Cavadore JC Schorderet-Slatkine S Okadaic acid and p13suc1 modulate the reinitiation of meiosis in mouse oocytes Mol Reprod Dev 1992 33 287 296 1333241 10.1002/mrd.1080330309

[121] Levesque JT Sirard MA Effects of different kinases and phosphatases on nuclear and cytoplasmic maturation of bovine oocytes Mol Reprod Dev 1995 42 114 121 8562045 10.1002/mrd.1080420115

[122] Sasseville M Côté N Guillemette C Richard FJ New insight into the role of phosphodiesterase 3A in porcine oocyte maturation BMC Dev Biol 2006 6 47 17038172 10.1186/1471-213X-6-47 PMC1617088

[123] Swain JE Ding J Brautigan DL Villa-Moruzzi E Smith GD Proper chromatin condensation and maintenance of histone H3 phosphorylation during mouse oocyte meiosis requires protein phosphatase activity Biol Reprod 2007 76 628 638 17182892 10.1095/biolreprod.106.055798

[124] Kimm-Brinson KL Ramsdell JS The red tide toxin, brevetoxin, induces embryo toxicity and developmental abnormalities Environ Health Perspect 2001 109 377 381 11335186 10.1289/ehp.01109377 PMC1240278

[125] Moon RT Morrill JB The effects of *Gymnodinium breve* lysate on the larval development of the sea urchin *Lytechinus variegatus* J Environ Sci Health 1976 11 673 683

[126] Steidinger KA Burklew MA Ingle RM The effects of *Gymnodinium breve* toxin on estuarine animals Marine Pharmacognosy Martin DF Padilla GM Academic Press New York, NY, USA 1973 179 202

[127] Miller MA Maternal transfer of lipophilic contaminants in Salmonines to their eggs Can J Fish Aquat Sci 1993 49 1405 1413

[128] Walker ST Fish mortality in the Gulf of Mexico Proc US Natl Mus 1884 6 105 109

[129] Taylor HF Mortality of fishes on the West Coast of Florida Report of the US Commissioner of Fisheries Bureau of Fisheries Document 848 Government Printing Office Washington, USA 1917

[130] Davis CC *Gymnodinium breve*: A cause of discolored water and animal mortality in the Gulf of Mexico Bot Gaz 1948 109 358 360

[131] Gunter G Williams RH Davis CC Smith FGW Catastrophic mass mortality of marine animals and coincident phytoplankton bloom on the west coast of Florida, November 1946 to August 1947 Ecol Monogr 1948 8 310 324

[132] Quick JA Henderson GE Evidences of new ichthyointoxicative phenomena in *Gymnodinium breve* red tides Proceedings of the First International Conference on Toxic Dinoflagellate Blooms LoCicero BR Massachusetts Science and Technology Foundation Wakefield, UK 1975 413 422

[133] Riley CM Holt SA Holt J Buskey EJ Arnold CR Mortality of larval red drum (*Sciaenops ocellatus*) associated with a *Ptychodiscus brevis* red tide Contrib Mar Sci 1989 31 137 146

[134] Forrester DJ Gaskin JM White FH Thompson NP Quick JA Henderson GE Woodard JC Robertson WD An epizootic of waterfowl associated with a red tide episode in Florida J Wildl Dis 1977 13 160 167 559108 10.7589/0090-3558-13.2.160

[135] Bossart GD Baden DG Ewing RY Roberts B Wright SD Brevitoxicosis in manatees (*Trichechus manatus latirostris*) from the 1996 epizootic: Gross, histologic and mmunohistochemical features Toxicol Pathol 1998 26 276 282 9547868 10.1177/019262339802600214

[136] Flynn KJ Irigoien X Aldehyde-induced insidious effects cannot be considered as a diatom defence mechanism against copepods Mar Ecol Prog Series 2009 377 79 89

[137] Miralto A Barone G Romano G Poulet SA Ianora A Russo GL Buttino I Mazzarella G Laabir M Cabrini M Giacobbe MG The insidious effect of diatoms on copepod reproduction Nature 1999 402 173 176

[138] Ianora A Miralto A Poulet SA Carotenuto Y Buttino I Romano G Casotti R Pohnert G Wichard T Colucci-D’Amato L Terrazzano G Smetacek V Aldehyde suppression of copepod recruitment in blooms of a ubiquitous planktonic diatom Nature 2004 429 403 407 15164060 10.1038/nature02526

[139] Buttino I De Rosa G Carotenuto Y Mazzella M Ianora A Esposito F Vitiello V Quaglia F La Rotonda MI Miralto A Aldehyde-encapsulating liposomes impair marine grazer survivorship J Exp Biol 2008 211 1426 1433 18424676 10.1242/jeb.015859

[140] Caldwell GS Olive PJW Bentley MG Inhibition of embryonic development and fertilization in broadcast spawning marine invertebrates by water-soluble diatom extracts and the diatom toxin 2-trans,4-trans-decadienal Aquatic Toxicol 2002 60 123 137 10.1016/s0166-445x(01)00277-6 12204592

[141] Caldwell GS Lewis C Olive PJ Bentley MG Exposure to 2,4-decadienal negatively impacts upon marine invertebrate larval fitness Mar Environ Res 2005 59 405 417 15603766 10.1016/j.marenvres.2004.06.005

[142] Tosti E Romano G Buttino I Cuomo A Ianora A Miralto A Bioactive aldehydes from diatoms block the fertilization current in ascidian oocytes Mol Reprod Dev 2003 66 72 80 12874802 10.1002/mrd.10332

[143] Ramsdell JS Zabka TS *In utero* domoic acid toxicity: A fetal basis to adult disease in the California sea lion (*Zalophus californianus*) Mar Drugs 2008 6 262 290 18728728 10.3390/md20080013 PMC2525490

[144] Scholin CA Gulland F Doucette GJ Benson S Busman M Chavez FP Cordaro J DeLong R de Vogelaere A Harvey J Haulena M Lefebvre K Lipscomb T Loscutoff S Lowenstine LJ Marin R 3rd Miller PE McLellan WA Moeller PD Powell CL Rowles T Silvagni P Silver M Spraker T Trainer V van Dolah FM Mortality of sea lions along the central California coast linked to a toxic diatom bloom Nature 2000 403 80 84 10638756 10.1038/47481

[145] Brodie EC Gulland FMD Greig DJ Hunter M Jaakola J Leger JS Leighfield TA van Dolah FM Domoic acid causes reproductive failure in California sea lions (*Zalophus californianus*) Mar Mam Sci 2006 22 700 707

[146] Goldstein T Zabka TS Delong RL Wheeler EA Ylitalo G Bargu S Silver M Leighfield T van Dolah F Langlois G Sidor I Dunn JL Gulland FM The role of domoic acid in abortion and premature parturition of California sea lions (*Zalophus californianus*) on San Miguel Island, California J Wildl Dis 2009 45 91 108 19204339 10.7589/0090-3558-45.1.91

[147] Lefebvre KA Powell CL Busman M Doucette GJ Moeller PDR Silver JB Miller PE Hughes MP Singaram S Silver MW Tjeerdema RS Detection of domoic acid in northern anchovies and California sea lions associated with an unusual mortality event Nat Tox 1999 7 85 92 10.1002/(sici)1522-7189(199905/06)7:3<85::aid-nt39>3.0.co;2-q 10647509

[148] Lefebvre KA Dovel SL Silver MW Tissue distribution and neurotoxic effects of domoic acid in a prominent vector species, the northern anchovy *Engraulis mordax* Mar Biol 2001 138 693 700

[149] Kvitek R Goldberg JD Smith GJ Doucette GJ Silver MW Domoic acid contamination within eight representative species from the benthic food web of Monterey Bay, California, USA Mar Ecol Prog Series 2008 367 35 47

[150] Ramsdell JS The molecular and integrative basis to domoic acid toxicity Phycotoxins: Chemisty and Biochemistry Botana L Blackwell Publishing Ames, IO, USA 2007 223 250

[151] Maucher JM Ramsdell JS Maternal-fetal transfer of domoic acid in rats at two gestational time points Environ Health Perspect 2007 115 1743 1746 18087593 10.1289/ehp.10446 PMC2137110

[152] Liu H Kelly MS Campbell DA Dong SL Zhu JX Wang SF Exposure to domoic acid affects larval development of king scallop *Pecten maximus* (Linnaeus, 1758) Aquat. Toxicol 2007 81 152 158 17178425 10.1016/j.aquatox.2006.11.012

[153] Pesando D Huitorel P Dolcini V Amade P Girard JP Caulerpenyne interferes with microtubule-dependent events during the first mitotic cycle of sea urchin eggs Eur J Cell Biol 1998 77 19 26 9808285 10.1016/S0171-9335(98)80098-8

[154] O’Brien ET Asai DJ Jacobs RS Wilson L Selective inhibition of cytokinesis in sea urchin embryos by low concentrations of stypoldione, a marine natural product that reacts with sulfhydryl groups Mol Pharmacol 1989 35 635 642 2725473

[155] Premakumara GA Ratnasooriya WD Tillekeratne LM Amarasekare AS Atta-Ur-Rahman Human sperm motility stimulating activity of a sulfono glycolipid isolated from Sri Lankan marine red alga *Gelidiella acerosa* Asian J Androl 2001 3 27 31 11250790

[156] Ratnasooriya WD Premakumara GA Tillekeratne LM Post-coital contraceptive activity of crude extracts of Sri Lankan marine red algae Contraception 1994 50291 299 10.1016/0010-7824(94)90074-4 7805379

[157] Williamson JE Carson DG de Nys R Steinberg PD Demographic consequences of an ontogenetic shift by a sea urchin in response to host plant chemistry Ecology 2004 85 1355 1371

[158] Ohta E Okada H Ohta S Kobayashi M Kitagawa I Horiike S Takahashi T Hosoya H Yamamoto K Ikegami S Malformation of immature starfish oocytes by theonellapeptolide Ie, a tridecapeptide lactone from a marine sponge *Petrosia* species, through disturbance of cortical F-actin distribution Biosci. Biotechnol. Biochem 2003 67 1908 1915 14519975 10.1271/bbb.67.1908

[159] Liu H Namikoshi M Meguro S Nagai H Kobayashi H Yao X Isolation and characterization of polybrominated diphenyl ethers as inhibitors of microtubule assembly from the marine sponge *Phyllospongia dendyi* collected at Palau J Nat Prod 2004 67 472 474 15043436 10.1021/np0304621

[160] Liu H Namikoshi M Akano K Kobayashi H Nagai H Yao X Seven new meroditerpenoids, from the marine sponge *Strongylophora strongylata*, that inhibited the maturation of starfish oocytes J Asian Nat Prod Res 2005 7 661 670 16087642 10.1080/1028602032000169604

[161] Bubb MR Senderowicz AM Sausville EA Duncan KL Korn ED Jasplakinolide, a cytotoxic natural product, induces actin polymerization and competitively inhibits the binding of phalloidin to F-actin J Biol Chem 1994 269 14869 14871 8195116

[162] Terada Y Simerly C Schatten G Microfilament stabilization by jasplakinolide arrests oocyte maturation, cortical granule exocytosis, sperm incorporation cone resorption, and cell-cycle progression, but not DNA replication, during fertilization in mice Mol Reprod Dev 2000 56 89 98 10737971 10.1002/(SICI)1098-2795(200005)56:1<89::AID-MRD11>3.0.CO;2-I

[163] Schatten G Schatten H Spector I Cline C Paweletz N Simerly C Petzelt C Latrunculin inhibits the microfilament-mediated processes during fertilization, cleavage and early development in sea urchins and mice Exp Cell Res 1986 166 191 208 3743654 10.1016/0014-4827(86)90519-7

[164] Spector I Shochet NR Blasberger D Kashman Y Latrunculins--novel marine macrolides that disrupt microfilament organization and affect cell growth: I. Comparison with cytochalasin D Cell Motil Cytoskeleton 1989 13 127 144 2776221 10.1002/cm.970130302

[165] Ohta E Uy MM Ohta S Yanai M Hirata T Ikegami S Anti-fertilization activity of a spirocyclic sesquiterpene isocyanide isolated from the marine sponge *Geodia exigua* and related compounds Biosci Biotechnol Biochem 2008 72 1764 1771 18603800 10.1271/bbb.80071

[166] Kobayashi M Kanzaki K Katayama S Ohashi K Okada H Ikegami S Kitagawa I Marine natural products. XXXIII. Theonellapeptolide IId, a new tridecapeptide lactone from the Okinawan marine sponge *Theonella swinhoei* Chem Pharm Bull (Tokyo) 1994 42 1410 1415 7923463 10.1248/cpb.42.1410

[167] Ikegami S Kobayashi H Myotoishi Y Ohta S Kato KH Selective inhibition of exoplasmic membrane fusion in echinoderm gametes with jaspisin, a novel antihatching substance isolated from a marine sponge J Biol Chem 1994 269 23262 23267 8083231

[168] Ohta S Kobayashi H Ikegami S Jaspisin: A novel styryl sulfate from the marine sponge, *Jaspis* species Biosci Biotechnol Biochem 1994 58 1752 1753

[169] Kato KH Takemoto K Kato E Miyazaki K Kobayashi H Ikegami S Inhibition of sea urchin fertilization by jaspisin, a specific inhibitor of matrix metalloendoproteinase Dev Growth Differ 1998 40 221 230 9572364 10.1046/j.1440-169x.1998.00011.x

[170] Uno M Ohta S Ohta E Ikegami S Callyspongins A and B: Novel polyacetylene sulfates from the marine sponge *Callyspongia truncata* that inhibit fertilization of starfish gametes J Nat Prod 1996 59 1146 1148

[171] Kobayashi M Shimizu N Kitagawa I Kyogoku Y Harada N Uda H Absolute stereostructures of halenaquinol and halenaquinol sulfate, pentacyclic hydroquinones from the Okinawan marine sponge *Xestospongia sapra*, as determined by theoretical calculation of CD spectra Tetrahedron Lett 1985 26 3833 3836

[172] Ikegami S Kajiyama N Ozaki Y Myotoishi Y Miyashiro S Takayama S Kobayashi M Kitagawa I Selective inhibition of membrane fusion events in echinoderm gametes and embryos by halenaquinol sulfate FEBS Lett 1992 302 284 286 1601136 10.1016/0014-5793(92)80460-x

[173] Ishihara H Calyculin A and okadaic acid: Inhibitors of protein phosphatase activity Biochem Biophys Res Commun 1989 159 871 877 2539153 10.1016/0006-291x(89)92189-x

[174] Smith GD Sadhu A Wolf DP Transient exposure of rhesus macaque oocytes to calyculin-A and okadaic acid stimulates germinal vesicle breakdown permitting subsequent development and fertilization Biol Reprod 1998 58 880 886 9546716 10.1095/biolreprod58.4.880

[175] Agius L Jaccarini V Ballantine JA Ferrito V Pelter A Psaila AF Zammit VA Photodynamic action of bonellin, an integumentary chlorin of *Bonellia viridis*, Rolando (Echiura, Bonelliidae) Comp Biochem Physiol B 1979 63 109 117 318394 10.1016/0305-0491(79)90242-6

[176] Pelter A Ballantine JA Ferrito V Jaccarino V Psaila AF Schembri PJ Bonellin, a most unusual chlorin J Chem Soc Chem Comm 1976 23 999 1000

[177] Cariello L De Nicola Giudici M Tosti E Zanetti L On the mechanism of action of bonellin on the sea urchin egg Gamete Res 1982 5 161 166

[178] De Nicola Giudici M Inhibition of motility by bonellin. II. Spermatozoa and embryos of sea urchin Acta Embryol Morphol Exp 1982 3 97 106 7184287

[179] Lakshmi V Saxena A Mishra SK Raghubir R Srivastava MN Jain RK Maikhuri JP Gupta G Spermicidal activity of bivittoside D from *Bohadschia vitiensis* Arch Med Res 2008 39 631 638 18760190 10.1016/j.arcmed.2008.06.007

[180] Simon-Levert A Arrault A Bontemps-Subielos N Canal C Banaigs B Meroterpenes from the ascidian *Aplidium aff densum* J Nat Prod 2005 68 1412 1415 16180826 10.1021/np050110p

[181] Simon-Levert A Aze A Bontemps-Subielos N Banaigs B Genevière AM Antimitotic activity of methoxyconidiol, a meroterpene isolated from an ascidian Chem Biol Interact 2007 168 106 116 17448456 10.1016/j.cbi.2007.03.004

[182] Sipkema D Franssen MC Osinga R Tramper J Wijffels RH Marine sponges as pharmacy Mar Biotechnol 2005 7 142 162 15776313 10.1007/s10126-004-0405-5 PMC7087563

[183] MarinLit A marine literature database maintained by the Marine Chemistry Group University of Canterbury Christchurch, New Zealand 1999

[184] Faulkner DJ Marine natural products Nat Prod Rep 2000 17 7 55 10714898 10.1039/a809395d

[185] Faulkner DJ Marine natural products Nat Prod Rep 2001 18 1 49 11245399 10.1039/b006897g

[186] Faulkner DJ Marine natural products Nat Prod Rep 2002 19 1 48 11902436 10.1039/b009029h

[187] Wijffels RH Potential of sponges and microalgae for marine biotechnology Trends Biotechnol 2007 26 26 31 18037175 10.1016/j.tibtech.2007.10.002

[188] Mayer AMS Gustafson KR Marine Pharmacology in 2005–6: Antitumor and Cytotoxic Compounds Eur J Cancer 2008 442357 2387 10.1016/j.ejca.2008.07.001 PMC2629923 18701274

[189] Glaser KB Mayer AMS A Renaissance in Marine Pharmacology: From Preclinical Curiosity to Clinical Reality Biochem Pharmacol 2009 78 440 448 19393227 10.1016/j.bcp.2009.04.015

[190] Mayer AMS Rodriguez AD Berlinck R Hamann MT Marine pharmacology in 2005–6: Marine Compounds with Antibacterial, Anticoagulant, Antifungal, Anthelmitic, Anti-inflammatory, Antiprotozoal, and Antiviral Activities; affecting the Cardiovascular, Endocrine, Immune and Nervous Systems and other Miscellaneous Mechanisms of Action Biochem Biophys Acta 2009 1790 283 308 19303911 10.1016/j.bbagen.2009.03.011 PMC2735450

[191] Kitagawa I Kobayashi M Kitanaka K Kido M Kyogoku Y Marine natural products, XII: On the chemical constituents of the Okinawan marine sponge Hymeniacidon aldis Chem Pharm Bull 1983 31 2321 2328

[192] Fedoreev SA Prokof’eva NG Denisenko VA Rebachuk NM Cytotoxic activity of aaptamines from suberitid marine sponges Pharm Chem J 1989 22 615 618

[193] Willis RH De Vries DJ BRS1, a C30 bis-amino, bis-hydroxy polyunsaturated lipid from an Australian calcareous sponge that inhibits protein kinase C Toxicon 1997 35 1125 1129 9248010 10.1016/s0041-0101(96)00218-8

[194] Jares-Erijman EA Sakai R Rinehart KL Crambescidins: New antiviral and cytotoxic compounds from the sponge *Crambe crambe* J Ore Chem 1991 56 5712 5715

[195] Berlinck RGS Braekman JC Daloze D Bruno I Riccio R Ferri S Spampinato S Speroni E Polycyclic guanidine alkaloids from the marine sponge *Crambe crambe* and Ca^++^ channel blocker activity of crambescidin 816 J Nat Prod 1993 56 1007 1015 8377012 10.1021/np50097a004

[196] Wang DZ Neurotoxins from marine dinoflagellates: A brief review Mar Drugs 2008 6 349 71 18728731 10.3390/md20080016 PMC2525493

[197] Kem WR Sea anemone toxins: Structure and action The Biology of Nematocysts Hessinger DA Lenhoff HM Academic Press New York, NY, USA 1988 375 405

[198] Kem WR Pennington MW Dunn BM Sea anemone polypeptide toxins affecting sodium channels. Initial structure-activity investigations Marine Toxins Origin, Structure and Molecular Pharmacology Hall S Stricharz G American Chemical Society Washington, USA 1990 279 289

[199] Norton TR Cardiotonic polypeptides from *Anthopleura xanthogrammica* (Brandt) and *A. elegantissima* (Brandt) Fed Proc 1981 40 21 25 6108877

[200] Honma T Shiomi K Peptide toxins in sea anemones: Structural and functional aspects Mar Biotechnol 2006 8 1 10 16372161 10.1007/s10126-005-5093-2 PMC4271777

[201] Llewellyn LE Sodium channel inhibiting marine toxins Prog Mol Subcell Biol 2009 46 67 97 19184585 10.1007/978-3-540-87895-7_3

[202] Castañeda O Sotolongo V Amor AM Stöklin R Anderson AJ Harvey AL Engström A Wernstedt C Karlsson E Characterization of a potassium channel toxin from the Caribbean sea anemone Stichodactyla helianthus Toxicon 1995 33 603 613 10.1016/0041-0101(95)00013-c 7660365

[203] Schweitz H Bruhn T Guillemare E Moinier D Lancelin J-M Béress L Lazdunski M Kalicludines and kaliseptine. Two different classes of sea anemone toxins for voltage-sensitive K+ channels J Biol Chem 1995 270 25121 25126 7559645 10.1074/jbc.270.42.25121

[204] Cotton J Crest M Bouet F Alessandri N Gola M Forest E Karlsson E Castañeda O Harvey AL Vita C Ménez A A potassium-channel toxin from the sea anemone *Bunodosoma granulifera*, an inhibitor for Kv1 channels. Revision of the amino acid sequence, disulfide-bridge assignment, chemical synthesis, and biological activity Eur J Biochem 1997 244 192 202 9063464 10.1111/j.1432-1033.1997.00192.x

[205] Gendeh GS Young LC de Medeiros LC Jeyaseelan K Harvey AL Chung MCM A new potassium channel toxin from the sea anemone *Heteractis magnifica*: Isolation, cDNA cloning, and functional expression Biochemistry 1997 36 11461 11471 9298966 10.1021/bi970253d

[206] Minagawa S Ishida M Nagashima Y Shiomi K Primary structure of a potassium channel toxin from the sea anemone *Actinia equina* FEBS Lett 1998 427 149 151 9613617 10.1016/s0014-5793(98)00403-7

[207] De Smet P Parys JB Callewaert G Weidema AF Hill E De Smedt H Erneux C Sorrentino V Missiaen L Xestospongin C is an equally potent inhibitor of the inositol 1,4,5-triphosphate receptor and the endoplasmic-reticulum Ca^2+^ pumps Cell Calcium 1999 26 9 13 10892566 10.1054/ceca.1999.0047

[208] Hirata Y Uemura D Halichondrins - antitumor polyether macrolides from a marine sponge Pure Appl Chem 1986 58 701 710

[209] Bai RL Paull KD Herald CL Malspeis L Pettit GR Hamel E Halichondrin B and homohalichondrin B, marine natural products binding in the vinca domain of tubulin: Discovery of tubulin-based mechanism of action by analysis of differential cytotoxicity data J Biol Chem 1991 266 15882 15889 1874739

[210] Bai R Cichacz ZA Herald CL Pettit GR Hamel E Spongistatin 1, a highly cytotoxic, sponge-derived, marine natural product that inhibits mitosis, microtubule assembly, and the binding of vinblastine to tubulin Mol Pharmacol 1993 44 757 766 8232226

[211] Koiso Y Morita K Kobayashi M Wang W Ohyabu N Iwasaki S Effects of arenastatin A and its synthetic analogs on microtubule assembly Chemico-Biol Interact 1996 102 183 191 10.1016/s0009-2797(96)03743-x 9021170

[212] Ter Haar E Kowalski RJ Hamel E Lin CM Longley RE Gunasekera SP Rosenkranz HS Day BW Discodermolide, a cytotoxic marine agent that stabilizes microtubules more potently than taxol Biochemistry 1996 35 243 250 8555181 10.1021/bi9515127

[213] Anderson HJ Coleman JE Andersen RJ Roberge M Cytotoxic peptides hemiasterlin, hemiasterlin A and hemiasterlin B induce mitotic arrest and abnormal spindle formation Cancer Chemother Pharmacol 1997 39 223 226 8996524 10.1007/s002800050564

[214] Mooberry SL Tien G Hernandez AH Plubrukarn A Davidson BS Laulimalide and isolaulimalide, new paclitaxel-like microtubule-stabilizing agents Cancer Res 1999 59 653 660 9973214

[215] Hood KA West LM Rouwé B Northocote PT Berridge MV Wakefield SJ Miller JH Peloruside A, a novel antimitotic agent with paclitaxel-like microtabule-stabilizing activity Cancer Res 2002 62 3356 3360 12067973

[216] Isbrucker RA Cummins J Pomponi SA Longley RE Wright AE Tubulin polymerizing activity of dictyostatin 1, a polyketide of marine sponge origin Biochem Pharmacol 2003 66 75 82 12818367 10.1016/s0006-2952(03)00192-8

[217] Kashman Y Groweiss A Shmueli U Latruncutin, a new 2-thiazolidinone macrolide from the marine sponge *Latrunculia magnifica* Tetrahedron Lett 1980 21 3629 3632

[218] Coue M Brenner SL Spector I Korn ED Inhibition of actin polymerization by latrunculin A FEBS Lett 1987 213 316 318 3556584 10.1016/0014-5793(87)81513-2

[219] Fusetani N Yasumuro K Matsunaga S Hashimoto K Mycalolides A–C, hybrid macrolides of ulapualides and halichondramide, from a sponge of the genus *Mycale* Tetrahedron Lett 1989 30 2809 2812

[220] Saito S Watabe S Ozaki H Fusetani N Karaki H Mycalolide, a novel actin depolymerizing agent J Biol Chem 1994 269 29710 29714 7961961

[221] Bubb MR Spector I Bershadsky AD Korn ED Swinholide A is a microfilament disrupting marine toxin that stabilizes actin dimers and severs actin filaments J Biol Chem 1995 270 3463 3466 7876075 10.1074/jbc.270.8.3463

[222] Anderluh G Macek P Cytolytic peptide and protein toxins from sea anemones (Anthozoa: Actiniaria) Toxicon 2002 40 111 124 11689232 10.1016/s0041-0101(01)00191-x

[223] Crnigoj Kristan K Viero G Dalla Serra M Maček P Anderluh G Molecular mechanism of pore formation by actinoporins Toxicon 2009, epub ahead of print 10.1016/j.toxicon.2009.02.026 19268680

[224] Amador ML Jimeno J Paz-Ares L Cortes-Funes H Hidalgo M Progress in the development and acquisition of anticancer agents from marine sources Ann Oncol 2003 14 1607 1615 14581267 10.1093/annonc/mdg443

[225] D’Incalci M Simone M Tavecchio M Damia G Garbi A Erba E New drugs from the sea J Chemother 2004 16 86 89 10.1179/joc.2004.16.Supplement-1.86 15688619

[226] Russo GL Ciarcia G Presidente E Siciliano RA Tosti E Cytotoxic and apoptogenic activity of a methanolic extract from the marine invertebrate *Ciona intestinalis* on malignant cell lines Med Chem 2008 4 106 109 18336328 10.2174/157340608783789121

